# Efficacy and safety of transumbilical laparoendoscopic single site surgery for mid-pregnancy ovarian cyst torsion: a retrospective analysis

**DOI:** 10.3389/fmed.2025.1649079

**Published:** 2025-09-17

**Authors:** Fei Wang, Yuqin Liu, Ru Wang, Yue Xing, Yongting Zhao, Wenxin Ha, Liehong Wang, Yufang Bai

**Affiliations:** ^1^College of Clinical Medicine, Qinghai University, Xining, China; ^2^Department of Gynaecology and Obstetrics, Qinghai Red Cross Hospital, Xining, China; ^3^Department of Gynaecology and Obstetrics, Qinghai University Affiliated Hospital, Xining, China

**Keywords:** TU-LESS, mid-pregnancy, ovarian cyst torsion, clinical efficacy, retrospective analysis

## Abstract

**Objective:**

To evaluate the efficacy and safety of transumbilical laparoendoscopic single site surgery (TU-LESS) in treating mid-pregnancy ovarian cyst torsion.

**Methods:**

We conducted a retrospective analysis of the clinical data of 54 mid-pregnancy patients who underwent open surgery for ovarian cyst torsion repositioning and cystectomy, and 31 patients who underwent TU-LESS at Qinghai Red Cross Hospital between January 2020 and January 2025. Key parameters analyzed included operative time, intraoperative blood loss, postoperative complications, hospital stay, postoperative first flatus time, preoperative and postoperative fetal heart rates, pregnancy outcomes, and postoperative visual analog scale (VAS) pain scores.

**Results:**

There were no significant differences between the two groups in terms of baseline characteristics such as age, BMI, tumor diameter, number of deliveries, history of pelvic surgery, pregnancy duration, duration of abdominal pain, or preoperative fetal heart rate. The TU-LESS group had significantly shorter operative times compared to the laparotomy group. No significant differences were observed in tumor pathology, intraoperative blood loss, or number of tumor ruptures. The TU-LESS group experienced shorter hospital stays, fewer postoperative complications, and lower VAS scores at 48 h post-operation compared to the laparotomy group. However, there were no significant differences between the groups in terms of time to first flatus, postoperative fetal heart rates, and VAS scores at 24 h post-operation.

**Conclusion:**

TU-LESS is a safe and feasible surgical method for treating mid-pregnancy ovarian cyst torsion and plays an important role in protecting maternal and fetal safety.

## Introduction

Ovarian cyst torsion during pregnancy is an acute abdomen that poses a significant threat to maternal and fetal safety, with an incidence approximately 2–3 times higher than in non-pregnant women, particularly in early pregnancy (1, 2). Factors such as uterine enlargement, altered pelvic anatomy, and fluctuating hormone levels during pregnancy result in atypical symptoms of ovarian cyst torsion, increasing diagnostic difficulty. Without timely intervention, severe complications such as ovarian ischemia and necrosis, infectious shock, and even maternal and fetal life endangerment may occur (3). In recent years, TU-LESS technology has been gradually promoted for benign gynecological diseases due to its minimally invasive and cosmetic advantages (4). TU-LESS is performed through a single umbilical incision, reducing postoperative pain and improving wound aesthetics (5). During mid-pregnancy, the uterus is not excessively enlarged, providing adequate space for surgical manipulation and creating a potential window for TU-LESS application. However, clinical research on TU-LESS for treating mid-pregnancy ovarian cyst torsion is currently limited, with a lack of comparative data with traditional open surgery. This study aims to explore the efficacy and safety of TU-LESS for mid-pregnancy ovarian cyst torsion by retrospectively comparing it with open surgery and evaluating preoperative, intraoperative, and postoperative outcomes to provide evidence-based recommendations for optimizing surgical management of pregnancy-related acute abdomen.

## Materials and methods

### General data

A retrospective analysis was conducted on the clinical data of 54 mid-pregnancy patients who underwent open surgery for ovarian cyst torsion repositioning and cystectomy, and 31 mid-pregnancy patients who underwent TU-LESS between January 2020 and January 2025 at Qinghai Red Cross Hospital. Before the surgery, the risks and advantages of the two surgical methods were explained in detail to the patient. The patient then chose the appropriate surgery based on their own condition. This retrospective study adhered to the principles of the Helsinki Declaration and received approval from the Ethics Committee of Qinghai Red Cross Hospital (LW-2024-155). All patients provided informed consent. Inclusion criteria: (1) gestational age between 14 and 27 + 6 weeks; (2) diagnosed ovarian cyst torsion requiring emergency surgical intervention; (3) preoperative malignant risk index (RMI) indicating a high probability of benign disease; (4) no preoperative symptoms of threatened abortion; (5) agreed to participate in the study and signed the consent form. Exclusion criteria: (1) preoperative suspicion of ovarian malignancy; (2) conversion to open surgery during the procedure; (3) severe pelvic adhesions; (4) accompanying other internal or external medical conditions.

### Surgical techniques

**Figure 1:**
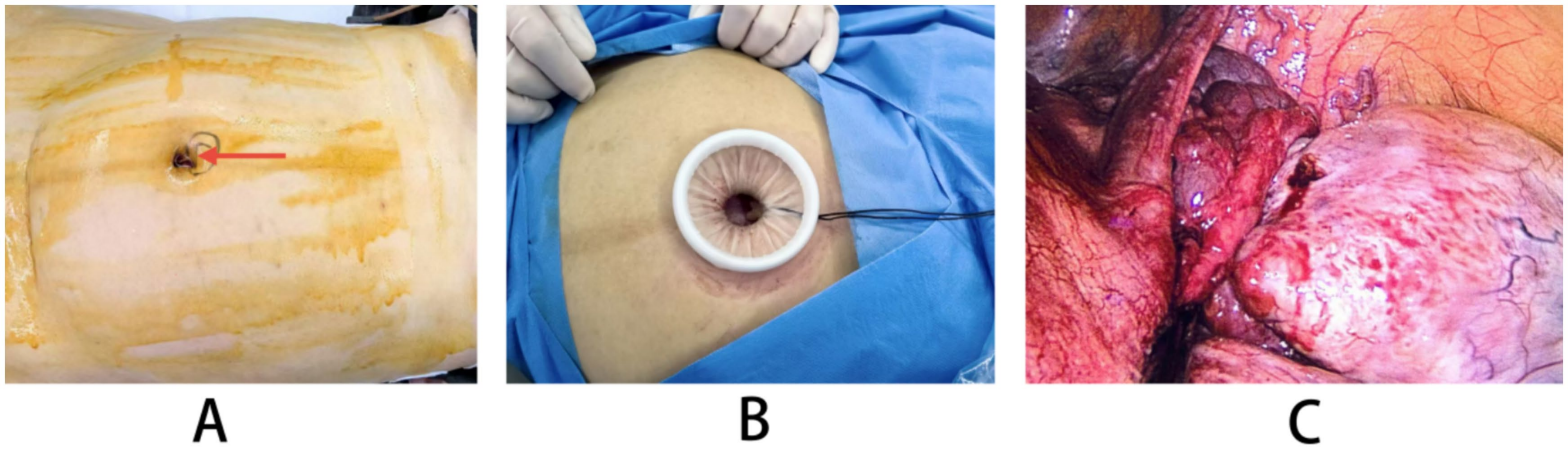
The surgical flowchart of TU-LESS. **(A)** The puncture site around the navel. **(B)** The layout diagram of the port device of TU-LESS. **(C)** Diagram of ovarian cyst torsion during surgery.

To minimize the impact of operator proficiency on perioperative outcomes, all procedures in this study were performed by a dedicated surgical team comprising 4 to 5 board-certified gynecologic oncologists. Each surgeon on the team has received specialized training and possesses over 5 years of extensive clinical experience in the field. The surgeries were consistently conducted by this fixed team, ensuring standardized operative techniques and reducing variability attributable to differing individual skill levels. All patients underwent rapid pathological diagnosis during surgery, which confirmed benign lesions. All patients met discharge criteria, including resumed semi-liquid diet, cessation of intravenous fluids, no signs of threatened abortion, well-healed wounds with no infection, and normal functional status upon re-examination. TU-LESS: Following general anesthesia, a 2 cm vertical incision was made at the umbilicus or 3–5 cm above the fundus, and a single-port laparoscopic device was inserted, maintaining pneumoperitoneum at 12–14 mmHg. The enlarged uterus was carefully observed to avoid injury. The location, size, and morphology of the cyst were examined, and the repositioned cyst was observed for ovarian necrosis. The cyst was separated from normal ovarian tissue, avoiding rupture, and the cyst wall was excised and placed in a specimen extraction bag. The ovarian incision was sutured with absorbable sutures, followed by rapid pathological examination. The pelvis and abdomen were irrigated with saline, and the puncture site was sutured. Fetal ultrasound examination and fetal heart monitoring were performed preoperatively and postoperatively. Laparotomy: A horizontal incision was made in the mid-abdominal area, followed by layered incision of subcutaneous tissue, opening of the peritoneum, and entry into the abdominal cavity. The cyst was repositioned and excised, and the ovarian incision was sutured, followed by rapid pathological examination. The pelvis and abdomen were irrigated with saline, and the incision was sutured in layers. Fetal ultrasound examination and fetal heart monitoring were performed preoperatively and postoperatively (Figure 1).

### Observation indicators

Preoperative general conditions (age, body mass index (BMI), tumor diameter, number of deliveries, history of pelvic surgery, pregnancy duration, duration of abdominal pain, preoperative fetal heart rate) and intraoperative conditions (surgical time, tumor pathology, intraoperative blood loss, number of tumor ruptures) were compared. Postoperative indicators were also assessed, including postoperative fetal heart rate, length of hospitalization, postoperative complications, duration of anal defecation, and VAS scores at 12 and 24 h post-operation.

### Statistical analysis

Statistical analysis was performed using IBM SPSS Statistics (Version 26.0, URL link: https://www.ibm.com/spss). Continuous variables were tested for normality, expressed as mean ± standard deviation if normally distributed, and compared using the t-test. Categorical variables were expressed as n (%) and compared using the χ2 test. A *p*-value < 0.05 was considered statistically significant.

## Results

### Preoperative general indicators comparison

No statistical differences were observed between the laparotomy and TU-LESS groups in terms of age, BMI, tumor diameter, number of deliveries, history of pelvic surgery, pregnancy duration, duration of abdominal pain, or preoperative fetal heart rate (*p* > 0.05) (see Table 1).

**Table 1:** Comparison of preoperative general information.

Group	Laparotomy (*n* = 54)	TU-LESS (*n* = 31)	t/χ^2^	*p* value
Age(years)	26.3 ± 4.1	25.9 ± 4.8	0.48	0.582
BMI(kg/m^2^)	22.7 ± 2.9	23.1 ± 2.5	1.43	0.537
Diameter of tumor(mm)	5.2 ± 1.1	5.0 ± 1.6	1.25	0.664
Pregnancy duration, n(%)				
≥14 weeks and <20 weeks	22 (40.7)	13 (41.9)	0.88	0.801
≥20 weeks and <28 weeks	32 (59.3)	18 (58.1)		
Number of deliveries, n(%)				
<2	38 (70.4)	22 (71.0)	0.62	0.853
≥2	16 (29.6)	9 (29.0)		
History of previous pelvic surgery, n(%)	8 (14.8)	5 (16.1)	2.43	0.387
Duration of abdominal pain(hours)	16.3 ± 2.4	15.7 ± 2.6	1.91	0.489
Preoperative fetal heart rate(bpm)	133.2 ± 8.1	135.0 ± 7.5	2.98	0.275

### Comparison of intraoperative outcomes between TU-LESS and open surgery

The TU-LESS group had significantly shorter operative times compared to the laparotomy group (*p* < 0.05). No significant differences were observed in tumor pathology, intraoperative blood loss, or number of tumor ruptures between the two groups (*p* > 0.05) (see Table 2).

**Table 2:** Comparison of intraoperative outcomes between TU-LESS and open surgery.

Group	Laparotomy (*n* = 54)	TU-LESS (*n* = 31)	t/χ^2^	*p* value
Pathological type, n(%)				
Serous cystadenoma	28 (51.9)	17 (54.8)	2.62	0.371
Teratoma	10 (18.5)	5 (16.1)		
Endometriotic cyst	13 (24.1)	7 (22.6)		
Others	3 (5.5)	2 (6.5)		
Surgical time(minutes)	67.3 ± 15.2	51.7 ± 7.3	12.36	0.013
Blood loss(mL)	25.3 ± 7.5	23.2 ± 6.4	1.07	0.695
Tumor rupture, n(%)	8 (14.8)	4 (12.9)	8.96	0.097

### Comparison of postoperative recovery and complications between TU-LESS and open surgery

The TU-LESS group experienced shorter hospital stays, fewer postoperative complications, and lower VAS scores at 48 h post-operation compared to the laparotomy group (*p* < 0.05). In the laparotomy group, there were 3 cases of surgical incision infections, occurring on the 4th, 5th, and 7th days post-operation, respectively. In the TU-LESS group, there was 1 case of surgical incision infection on the 5th day post-surgery. After anti-infection treatment, all patients recovered to normal. In the laparotomy group, 5 cases developed threatened abortion post-operation, whereas in the TU-LESS group, 2 cases developed threatened abortion. After anti-contraction treatment to preserve the pregnancy, 1 case of abortion occurred in the laparotomy group, while the remaining patients delivered normally. No significant differences were observed between the groups in terms of time to first flatus, postoperative fetal heart rate, and VAS scores at 24 h post-operation (*p* > 0.05) (see Table 3).

**Table 3:** Comparison of postoperative recovery and complications between TU-LESS and open surgery.

Group	Laparotomy (*n* = 54)	TU-LESS (*n* = 31)	t/χ^2^	*p* value
Postoperative fetal heart rate(bpm)	132.5 ± 6.4	134.3 ± 5.7	2.87	0.293
Length of hospitalization(days)	7.3 ± 1.4	3.8 ± 1.3	21.36	0.002
Postoperative complication, n(%)				
Surgical incision infections	3 (7.3)	1 (2.9)		0.044
Threatened abortion	5 (12.2)	2 (5.7)	9.35	
Abortion	1 (2.4)	0		
Time to first flatus(hours)	19.2 ± 3.9	20.3 ± 3.1	4.01	0.137
VAS score at 12 h postoperatively	3.3 ± 1.0	3.1 ± 0.8	1.46	0.534
VAS score at 24 h postoperatively	2.6 ± 1.5	1.6 ± 1.1	12.28	0.015

## Discussion

Ovarian cyst torsion is a significant acute abdomen complication during pregnancy, posing severe threats to maternal and fetal health (6–8). Its incidence rate markedly increases during pregnancy, rising 2–3 times compared to non-pregnant periods (9). This elevation is primarily attributed to the enlargement of the uterus during pregnancy, which alters the position of the ovaries, and changes in hormone levels during pregnancy that encourage cyst growth and displacement of their center of gravity (10, 11). If torsion occurs and timely intervention is not undertaken, it can lead to severe consequences such as ovarian necrosis, rupture, and even miscarriage (12). Although traditional open surgery effectively addresses acute conditions, it poses considerable interference with the gravid uterus, has a prolonged postoperative recovery period, and carries a high risk of complications (13, 14). With the advent of minimally invasive techniques, TU-LESS has been widely adopted in gynecological surgery due to its unique advantages (15). However, its application value in managing acute abdomen during pregnancy, particularly in mid-pregnancy, requires further exploration.

In this study, we compared perioperative indicators and maternal-fetal outcomes between TU-LESS and open surgery, revealing significant advantages of the TU-LESS group. Specifically, the TU-LESS group had significantly shorter operative times than the open surgery group. Notably, the TU-LESS group also demonstrated superior postoperative outcomes in terms of pain scores (VAS) at 48 h, hospitalization duration, and complication rates. This highlights the rehabilitation benefits of minimally invasive surgery. Regarding maternal-fetal safety, there was no significant difference in postoperative fetal heart rates between the two groups, indicating that TU-LESS does not increase the risk of acute fetal stress. Our study results show that the operative time in the TU-LESS group was significantly shorter than in the open surgery group, challenging the traditional belief that single-port procedures increase operation time. This advantage stems from consolidated instrument control and a shorter surgical pathway: the single-port system avoids instrument interference associated with multi-port operations, and the navel, as a natural scar, provides a direct approach to the elevated attachments during pregnancy (16, 17). It is particularly noteworthy that shortening operative time during pregnancy is crucial for reducing anesthesia exposure and surgical stress, directly impacting maternal-fetal safety. Regarding intraoperative key safety indicators, there were no significant differences between the two groups in blood loss or tumor rupture rates, confirming that TU-LESS offers a comparable safety assurance to traditional open surgery in managing acute abdomen during pregnancy. The TU-LESS group’s postoperative hospital stay was significantly reduced, averaging 3.5 days less than the open surgery group. Additionally, the 48-h VAS score was significantly lower in the TU-LESS group, attributable to minimal nerve injury from the single umbilical incision and the avoidance of muscle layer suturing. Postoperative complication control is a key dimension for assessing surgical safety. In this study, the open surgery group had three cases of wound infection, while the TU-LESS group had only one case; although this is not statistically significant, studies indicate that the single incision of TU-LESS reduces contamination pathways. Moreover, the navel, with its rich blood supply and distance from the uterus operational area, effectively lowers the risk of cross-infection. Regarding pregnancy outcomes, the open surgery group had five cases of threatened miscarriage, while the TU-LESS group had only two cases, both of which successfully maintained the pregnancy. Open surgery involves larger incisions and more extensive uterine manipulation, leading to increased prostaglandin release; TU-LESS, utilizing transumbilical single-port operations, significantly reduces uterine traction. Fetal safety is the core concern of surgical procedures during pregnancy. Our study results show no significant differences in postoperative fetal heart rates between the two groups, indicating that TU-LESS maintains fetal hemodynamic stability as effectively as open surgery. In mid-pregnancy, the uterine fundus rises to the umbilical level; TU-LESS can employ an open technique to layer-by-layer cut the umbilical region under direct vision and insert the Port system, avoiding the risks associated with blind puncture (18). This technique’s advantages are particularly pronounced in pregnancy: it avoids inadvertent injuries caused by an enlarged gravid uterus and reduces repeated puncture injuries from failed pneumoperitoneum needle attempts (19). Furthermore, the TU-LESS postoperative incision can be perfectly concealed within the navel depression, which, for young pregnant women concerned about physical aesthetics, not only addresses appearance but also significantly reduces postoperative anxiety, enhancing treatment compliance (20, 21).

Limitations and Future Directions: Limitations and Future Directions: Although this study did not observe cases requiring conversion from TU-LESS to open surgery, nor did it report adverse fetal effects related to the pneumoperitoneum pressure (maintained at 12–14 mmHg for about 1 h) or CO_2_ insufflation, these remain recognized potential risks inherent to the procedure. The continuous pneumoperitoneum and CO_2_ exposure could theoretically impact fetal hemodynamics and acid–base balance, underscoring the need for cautious intraoperative monitoring. While our findings indicate TU-LESS is safe and effective within the current sample, these physiological concerns warrant further systematic evaluation. Future studies with larger cohorts and multicenter collaboration should specifically assess fetal outcomes related to pneumoperitoneum parameters and the incidence and management of conversion cases to open surgery. Additionally, extending follow-up periods will help elucidate any long-term maternal and neonatal sequelae. Developing standardized protocols and operator training programs for TU-LESS during pregnancy is essential to mitigate these potential risks and optimize surgical results. In addition, this study’s retrospective single-center design and relatively small sample size impose inherent limitations on the generalizability of the findings. Being confined to a single institution, the results may reflect center-specific surgical practices and patient characteristics, which could limit the applicability of the conclusions to broader populations. Furthermore, the retrospective nature of the study introduces potential biases in data collection and analysis. Prospective, multicenter studies with larger cohorts are warranted to validate and extend these findings, thereby enhancing the external validity and robustness of evidence regarding the safety and efficacy of TU-LESS in managing ovarian cyst torsion during mid-pregnancy.

## Conclusion

This study demonstrates that TU-LESS for treating mid-pregnancy ovarian cyst torsion offers significant advantages over open surgery, including shorter operative time, reduced postoperative pain, shorter hospitalization, and fewer complications. These benefits suggest that TU-LESS may provide certain safety advantages; however, safety profiles should be interpreted cautiously and require further validation through larger, prospective studies. Overall, TU-LESS represents a promising advancement in minimally invasive surgery during pregnancy. With continued technical refinement and standardized protocols, TU-LESS has the potential to further minimize surgical trauma and improve patient quality of life, offering an optimized approach for managing gynecological acute abdomen in pregnancy.

## Data Availability

The original contributions presented in the study are included in the article/supplementary material, further inquiries can be directed to the corresponding authors.
